# The role of models as a decision-making support tool rather than a guiding light in managing the COVID-19 pandemic

**DOI:** 10.3389/fpubh.2022.1002440

**Published:** 2022-12-01

**Authors:** Adi Niv-Yagoda, Royi Barnea, Efrat Rubinshtein Zilberman

**Affiliations:** ^1^School of Health Systems Management, Netanya Academic College, Netanya, Israel; ^2^Sackler Faculty of Medicine, Tel Aviv University, Tel Aviv, Israel; ^3^Assuta Health Services Research Institute, Assuta Medical Centers, Tel-Aviv, Israel; ^4^Hillel Yaffe Medical Center, Hadera, Israel

**Keywords:** COVID-19, models, health policy, evidence based decision-making, public health

## Abstract

Reference scenarios based on mathematical models are used by public health experts to study infectious diseases. To gain insight into modeling assumptions, we analyzed the three major models that served as the basis for policy making in Israel during the COVID-19 pandemic and compared them to independently collected data. The number of confirmed patients, the number of patients in critical condition and the number of COVID-19 deaths predicted by the models were compared to actual data collected and published in the Israeli Ministry of Health's dashboard. Our analysis showed that the models succeeded in predicting the number of COVID-19 cases but failed to deliver an appropriate prediction of the number of critically ill and deceased persons. Inherent uncertainty and a multiplicity of assumptions that were not based on reliable information have led to significant variability among models, and between the models and real-world data. Although models improve policy leaders' ability to act rationally despite great uncertainty, there is an inherent difficulty in relying on mathematical models as reliable tools for predicting and formulating a strategy for dealing with the spread of an unknown disease.

## Introduction

By its very nature, health is a statistical science replete with uncertainty, which is particularly high in certain situations such as pandemics. Hence over the years, and in various situations, decision makers in different countries have sought the help of reference scenarios and models to predict disease spread as a tool for effective ways to prevent it and for formulating tailored health policies (1–3). This was also the case when the magnitude of local infection with COVID-19 in the Chinese province of Wuhan came to light and morbidity spread to other countries until the WHO declared it a pandemic (4).

With the increasing reports about the spread of the disease, and like many countries worldwide, Israel adopted the recommendations of the WHO and began to take behavioral-social preventive measures in order to slow down and reduce the rate of COVID-19 spread. The measures described below were incorporated into Israel's health policy, some of them were unique to Israel.

### Preventive measures taken before the first COVID-19 wave

In January 2020, health institutions were instructed to be vigilant regarding individuals who returned from China and showed symptoms of illness (fever, cough, etc.), and to increase awareness among medical teams, and the importance of protecting the treating staff.

From the end of January 2020, an order established the mandatory isolation in a dedicated hospital setting of any person who returned from China showing symptoms of illness. The order also stated that forced isolation can be carried out for a person who shows resistance to voluntary isolation. At the same time, flights arriving from China were prohibited from landing in Israel, and later the entry of tourists to Israel from East-Asian countries (Japan, Hong Kong, South Korea, Thailand, etc.) were prohibited. In February 2020, airline routes from East Asian countries to Israel were closed, followed by routes from countries with excess morbidity – Italy and Spain. Finally, in the second week of March, air traffic to Israel was significantly reduced to the point of almost complete closure of Israel's air, land and sea borders (with the exception of rescue flights to repatriate Israeli residents abroad). At the same time (February 2020) a mandatory 14-day home isolation period was established – first for those returning from China and other East Asian countries, then for those returning from Italy, Spain, France, Germany, Switzerland and Austria. Finally, in the second week of March, the obligation of home isolation was extended to all those entering the State of Israel.

The first COVID-19 patient was diagnosed in Israel on February 23, 2020. The turning point of disease spread occurred in the second week of March 2020, when an exponential increase in new cases was observed in the country. Between March 2020 and March 2022, Israel faced five COVID-19 waves which resulted in over 4 million cases and over 10,000 deaths.

### Measures taken during the first wave

In March gatherings and mass events were gradually decreased from 2,000 to 100 participants and were then banned. Places of recreation and leisure as well as workplaces were closed (except for those defined as essential workers – 15% activity in the economy). On March 17, 2020, lockdown was declared. The education system and universities were shut down. Public transportation was significantly reduced, and residents were instructed not to leave their house, except for essential needs (food, medication and essential work). Later, the citizens were instructed not to go further than 100 meters of their place of residence (except for essential needs) and the gathering of more than two people who do not leave in the same household was prohibited. These instructions were anchored by the government as emergency regulations on March 25, 2020. In an unprecedented manner, in March 2020 the Israeli government decided to make use of advanced technological means of cellular tracking in order to enforce the obligation of isolation. In addition, and as a tool for epidemiological investigation, the General Security Service was authorized (under emergency regulations) to collect and process detailed information about the location and movement routes of people who were diagnosed with COVID-19 from 14 days before the diagnosis, with the aim of identifying contacts and isolating possible infection circles. Once the system was activated, individuals began to receive proactive messages about being in the vicinity of a confirmed COVID-19 patient without revealing the details of the patient himself. This move to track individuals by cell phones has received a lot of public criticism on the grounds of a severe and disproportionate violation of basic rights, including the right to privacy.

In the second week of April 2020, due to fear of gatherings and contagion during the Jewish holiday Passover, it was decided to tighten lockdown and move to a temporary state of curfew for the entire days of the holiday. Police and judicial forces were deployed across the country while blocking mobility between cities and strictly enforcing those who violated the curfew conditions (among other things by imposing fines between NIS 500 and NIS 5,000). Lockdown restrictions were gradually lifted in May and June 2020.

### Measures taken during the second wave

On September 18, 2020, a second lockdown for 21 days was announced in Israel. A few days later, it was decided to take further steps to tighten the lockdown. A differential program called “The Traffic Light”, which comprised classification of cities according to morbidity levels, was instated. On October 17, 2020, lockdown ended, except for cities defined as 'red cities' under the Traffic Light program.

### Measures taken during the third wave

On December 27, 2020, due to a renewed increase in morbidity, a third lockdown was announced. Unlike the previous two lockdowns, this time the Israeli government decided not to close educational institutions and the scope of work in the private sector was only reduced to 50%.

### Measures taken during the fourth and fifth waves

From June 2021, after elections and the establishment of a new government, Israel decided to move from a policy of lockdowns in response to increased morbidity to softer preventive measures. These included encouraging vaccinations, reducing gatherings, providing green certificates to vaccinated individuals which allowed them to enter shops and other public places, and monitoring morbidity.

### The use of models to predict morbidity and mortality

Reference scenarios are based on mathematical models used by public health experts to study infectious diseases. Such scenarios have many advantages, but they also present significant challenges, disadvantages and are sometimes even misleading. Starting in March 2020 experts from various fields began publishing in the public domain predictions on the “behavior” of the Severe Acute Respiratory Syndrome Corona Virus 2 (SARS-CoV-2) and the expected morbidity, mortality, and duration of the pandemic. These scenarios ranged from very optimistic to pessimistic ones. The models were designed to assist decision makers in dealing with core questions about the pandemic, such as the expected daily number of infections, the expected burden on hospitals, and policy implications. The predictions were based on mathematical calculations, hypotheses and assumptions, but on very little reliable information.

The Israeli Ministry of Health began to work with several reference scenarios formulated based on various assumptions at a relatively early stage. Most of the models were based on the Susceptible-Infectious-Recovered (SIR) model for studying epidemic spread as first published in 1927 by Kermack and McKendrick (5). Although this mathematical model has evolved and developed over the years, its basic principles have not changed. Thus, most models used for predicting COVID-19 disease spread have used the basic reproduction number (R0) as a tool to reflect the intensity of epidemic spread and have applied the principles of population classification into 3 groups: susceptible, infected and recovered. R0 is the mean number of secondary cases an infected person can cause in a population where there is no immunity. R0 was calculated by comparing the number of infected individuals in a given week with the number of infected individuals in the previous week. This index is greatly affected by a range of factors that can be influenced both by the characteristics of the population and by preventive measures. Although R0 is an important tool for developing theoretical models, its effectiveness in predicting the spread diseases was not tested prior to the COVID-19 pandemic. The most optimistic scenario (R0 = 1.2) estimated that there would be 108,000 critically ill patients with COVID-19 in Israel and that 8,600 would die of the disease, while the most pessimistic one (R0 = 2) predicted that there would be 270,000 critically ill patients and that 21,600 would die (The definition of critical cases was based on the Israeli Ministry of Health's definition, which included oxygen saturation levels below 94% as the main criteria). These scenarios were first presented to decision-makers and later to the public without proper and balanced mediation of the information, which led to the escalation in national anxiety and panic. At first the panic was translated into a rare public collaboration with government directives, but as time went on and the actual number of cases, critically ill patients and deaths turned out to be fundamentally different, the scenarios and forecasts became a double-edged sword as public trust and cooperation began to falter.

Although reference scenarios are based on a seemingly objective mathematical models, they often embody a variety of subjective assumptions due to the uncertainty inherent in unfamiliar morbidity. Models can only be useful in the context of imperfect information. In the absence of reliable information, some of these assumptions are influenced by the modelist's personal and/or professional perceptions of the characteristics of the disease, its future behavior, and the behavior of the public.

Assessment of gaps between models and real-world data, can assist policy makers in adopting an informed, data-based approach and can advance knowledge-based decision-making processes when dealing with subsequent crises. We examined the various reference scenarios that were presented to decision-makers in Israel and tried to estimate their quality compared to the actual data collected.

### Study data and methods

Although many prediction models were developed during the COVID-19 pandemic, we analyzed the 3 major models that served as the basis for policy making. Model data used in this analysis is public information that was made available by the three model teams at different timepoints during the pandemic on social media, television, press, official social networks, and as presentations presented to health policy leaders.

The models were analyzed anonymously. Due to the multiplicity of existing data in the field, we focused on comparing the predicted number of confirmed COVID-19 patients, patients in critical condition and deaths to the actual data collected and published in the Israeli Ministry of Health's dashboard.

For convenience, we defined the five COVID-19 waves in Israel:

First wave: March–May 2020

Second wave: June–November 2020

Third wave: December 2020–May 2021

Fourth wave: June–November 2021

Fifth Wave: December 2021–February 2022.

For the purpose of this study, a model forecast beyond ±10% of the ‘real world data’ is over/under estimating.

## Study results

**Figure 1:**
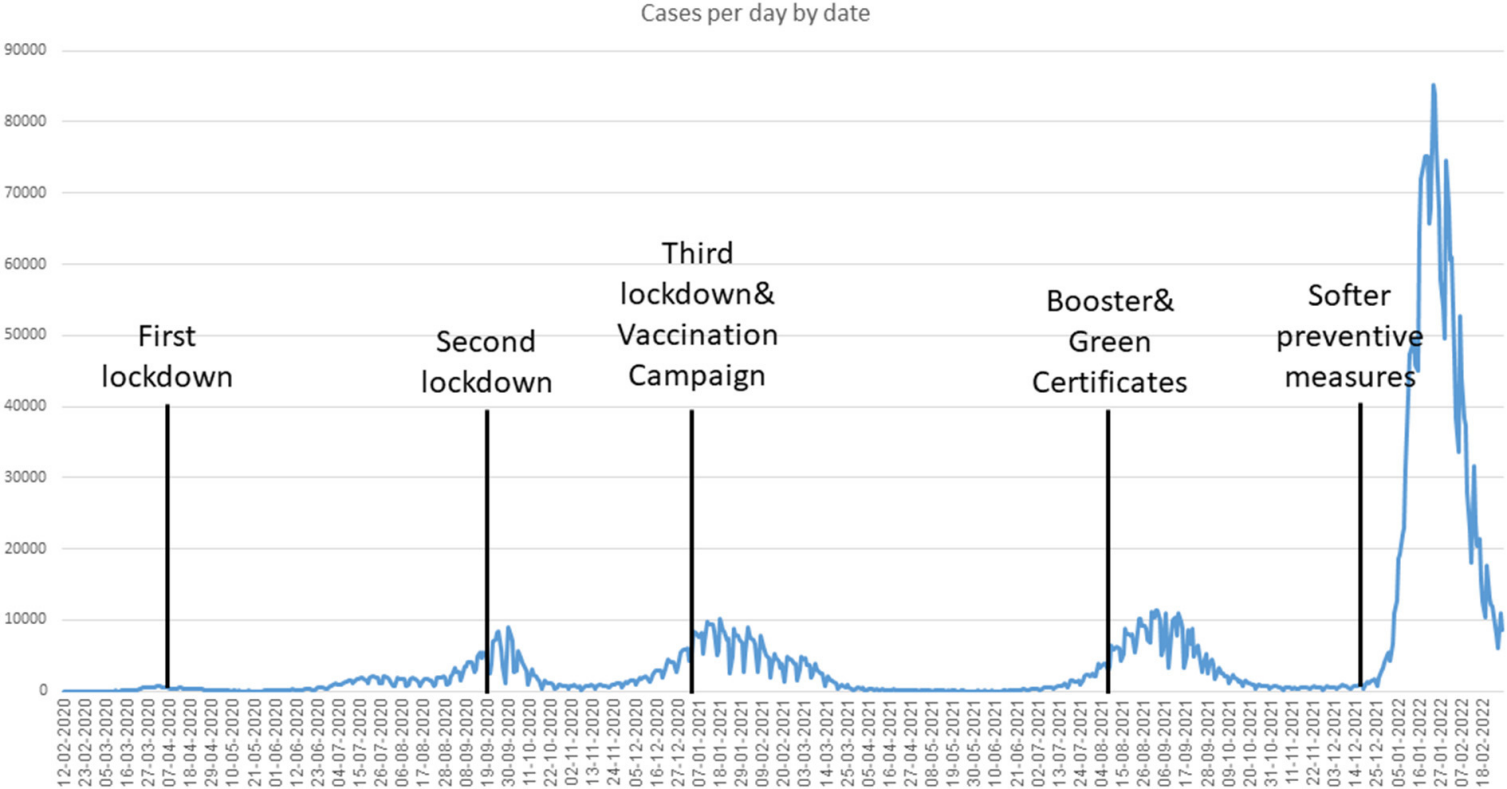
Number of confirmed COVID-19 infections per day in Israel (March 2020–February 2022).

The progress of the number of new cases in Israel is presented in Figure 1. Table 1 presents a comparison of the models and real-world data published by the Israeli Ministry of Health. It is important to note that the table is based on publications of the models and thus reflect different outcomes, which differ between the models. For example, while some of the models presented the cumulated number of new cases, others focused on the highest (peak) number of new cases and did not provided forecast of the cumulated number of cases for the entire wave.

**Table 1:** Comparison of models and real-world data, Israel March 2020–February 2022.

	**Ministry of health data**	**Team 1**	**Team 2**	**Team 3**
**Model description**		Combined models based on the infection characteristics: Survival analysis Kaplan Meier) and Cox proportional hazards model Aalen johsnaen estimator self-developed model with Monte Carlo simulation	Age-of-Infection model: number of cases has Poisson distribution characteristics with dynamic expectation	Short-term modeling (nowcasting) combined with other stochastic models (agent-based models)
**First wave: March-May 2020**				
Confirmed COVID-19 cases Tests	Peak: 17,047 (peak 724/day) No data	–	18,000–193,000	576,000–1,440,000
Critically ill	192	224,366	108,000–270,000	108,000–270,000
Dead	289	46,772	8,600–21,600^*^	8,600–21,600^*^
**Second wave: June—November 2020**				
Confirmed COVID–19 cases Tests	319,921 (Peak 9,051/day) 3,362,484		298,000	7,500–9,200 peak
Critically ill	495	976–1,193 Weekly forecast	critically and moderately ill: 9,550	820
Dead	2,596	From March 20: 8,361–10,534 (8,000–10,200 for the second wave alone)	1300	, 1,600
**Third wave: December 2020—May 2021**				
Confirmed COVID–19 cases Tests	Peak: 10,123 502819 (peak 10,123/day) 10,375,126	16,700–22,300 Depending on sensitivity analysis for vaccine efficiency and initial R	Differs between different scenarios (strict lockdown yes/no) 6,120–10,000	4,000–8,000 peak
Critically ill	1,193	1,700–2,400	Differs between different scenarios (strict lockdown yes/no) and on vaccination status 600–1,474 (lockdown y/n) 1,340–3,230 (vaccine y/n)	2,539–6,834 (vaccine y/n)
Dead	3,541	2,450–2,700	Differs between different scenarios (strict lockdown yes/no) and on vaccination status 1,250–3,085 (lockdown y/n)	4,500–5,700 On average, depending on preventive actions
**Fourth wave: June—November 2021**				
Confirmed COVID–19 cases Tests	50,4587 (Peak 11,346/day) 18,437,810	11,000 peak	Mainly children and unvaccinated adolescents	9,000 peak
Critically ill	766	850	Varied based on the vaccination status and based on age. 1000 (with booster)–2,500 (no booster)	2,000
Dead	1,782	–	200–1,400 Depends on the rate of the vaccination campaign (combined model teams 2 and 3)	1,500 according to mortality model
**Fifth wave: December 2021—February 2022**				
Confirmed COVID–19 cases Tests ^**^	2,293,405 (Peak 85,192/day) 18,369,900	45,000/day	4,000,000	73,000–46,000/day
Critically ill	1,255	800–1,000	1,250–2,750	700–1,566
Dead	2,042			100/week

* Teams 2 and 3 worked together at the beginning of the pandemic, joining forces in order to provide scientific assumptions to policy makers. As a result, the same prediction was provided by both teams. Later on, as these teams separated, differences in forecasts were observed.

** The number of tests during the fifth wave includes both PCR tests and Rapid (Antigen) tests.

### Models description

There were inherent differences between the different models as each model uses different techniques and relies on specific characters and scientific approaches. Severe cases were defined by the models based on the MOH definition (saturation levels, intensive care units). The models evolved over time to include additional variables such as vaccination status of various populations, the effect of various stringency of lockdowns, and other preventive actions. The purpose of team number 1 was to model the risk for the collapse of the health system. This was achieved by modeling the chance for hospitalization and death for each new case. The team developed a new model which was used with Monte Carlo simulation and combined models based on the characteristics of the infected individuals. The model included a survival analysis with Kaplan Meier and a Cox proportional hazards model. Team number 2 used an Age-of-Infection model. It uses the number of cases which has characteristics of Poisson distribution with dynamic expectation. Team number 3 performed short-term modeling (nowcasting) combined with other stochastic models (agent-based models) that examined the individual and followed the course of the exposure (infected, hospitalized, deceased, etc). The model presented a forecast of the presence and the near future and aimed to identify important parameters and provide predictions based on these parameters.

#### The first wave: March–May 2020

The models' overestimation of morbidity in comparison to actual data is highly evident in the analysis of First-Wave data. The predicted cumulative number of confirmed cases for this wave was up to 100 times higher than the actual number of confirmed cases. A similar trend was observed for the comparison between the predicted and actual number of deaths and critically ill patients. The largest gap between predicted and actual data was observed in Team 3's model. Team 3 predicted that the number of confirmed cases would be 50–100 times higher than actual data. Interestingly, Teams 2 and 3 predicted the same range of deaths, probably because they used R0 as the main explanatory variable in their sensitivity analyses. Team 1's number of predicted deaths was 161 times higher than the actual data (46,772 vs. 289).

#### The second wave: June–November 2020

Ministry of Health data showed that at the Second Wave's peak, at the end of September 2020, there were 9,051 confirmed cases and the cumulative number of confirmed cases for the entire wave was 319,921. This number corresponds with the models' predictions. However, all three models provided a significant overestimation of the number of critically ill patients (495 at the wave's peak). Teams 1 and 3 overestimated this number by 2–3 times. Team 2's estimation was even higher, but this team combined the number of patients in moderate and critical condition. Interestingly, Teams 2 and 3 predicted a significantly lower number of deaths compared to the actual data, while Team 1 overestimated the number of deaths by more than 100%. It is important to note that Team 1 only predicted the number of new patients in critical condition and did not provide predictions for the number of confirmed cases. Therefore, it is difficult to assess the effectiveness of the model in predicting disease spread (in terms of new cases) during the second wave.

#### The third wave: December 2020–May 2021

In the Third Wave Team 2 provided the closest prediction to actual data – both in the number of confirmed cases and in the number of deaths. This team tried to evaluate the effectiveness of strict lockdown alongside the vaccination campaign; therefore, it made a relatively careful estimation (under estimation) of the number of confirmed cases. Actual data showed an initial increase in the number of confirmed cases which decreased slowly. This trend was not predicted by the model. The models that were relatively accurate in predicting the number of confirmed cases in the second wave, made significant overestimations in the third wave, probably due to the start of the vaccination campaign.

#### The fourth wave: June–November 2021

The fourth wave was characterized by the spread of the delta strain of the virus, along with a decline in immune defense. Most teams were successful in predicting the number of confirmed cases as well as the number of deaths, with only about 10% deviation between the model and actual data. In contrast, the models, and particularly that of Team 3, overestimated the number of critically ill patients (2000 predicted vs. 766 actual). Team 2 developed a model which considered the vaccination booster as a crucial parameter in predicting the number of critically ill (1000–2500). However, both predicted values presents a large-scale overestimation. It is possible that the gradual decline in immune protection may have benefited the models by reducing the gap between the actual number of critically ill patients and their predicted number. Although the models did not account for decreased immune protection but rather provided their prediction based on the number of confirmed cases, the decrease in immune protection led to an increase in the number of critically ill patients, bringing it closer to the predicted number.

#### The fifth wave: December 2021–February 2022

The rapid spread of the omicron variant, which manifested in a very high number of daily confirmed cases (up to a peak of 85,000 confirmed cases per day and a total of about 2.3 million confirmed cases in this wave), resulted in high variance among the models regarding the number of confirmed cases and the rate of increase in cases. While Team 1 predicted an average of 45,000 confirmed cases per day, Teams 2 and 3 predicted a cumulative number of confirmed cases that was double compared to the actual data: Team 2 estimated that there would be 4 million confirmed cases (vs. 2.3 million cases that were actually confirmed) and Team 3 predicted that there would be 73,000–146,000 confirmed cases per day. There was considerable variation among the models in the predicted number of critically ill patients: while Team 1 predicted a lower number of critically ill patients than the actual number, Team 2 overestimated their number by over 100%, and Team 3, which provided gloomy predictions for most of the waves, accurately predicted the number of critically ill patients in the fifth wave. It is also important to note that due to multiple contradictory information on the severity of disease caused by the Omicron strain, information on the predicted number of deaths was not published by the modeling team. Instead, they chose to focus on the number of newly confirmed cases and the number of critically ill patients.

## Discussion

### Models' evaluation

From the day it was identified, SARS-CoV-2 has proven its ability to surprise healthcare systems worldwide. Tthroughout the COVID-19 pandemic in Israel, the reference models overestimated morbidity in comparison to actual data from the Ministry of Health. High variance was observed both among the models and for each model over time. Each modeling team chose to focus on a different outcome. For example, in later waves Team 1 moved to focus on patients in critical condition while Team 3 continued to provide predictions regarding the number of confirmed COVID-19 cases.

An examination of the various models by time shows that in most cases all teams overestimated the number of confirmed COVID-19 cases and critically ill patients with better accuracy in later stages of the pandemic. This is probably a consequence of the lack of uniformity in the knowledge available to the various modelers during the pandemic. The models usually used the R0 number as the primary variable for predicting the spread of disease. Considering the evolution and variability of SARS-CoV-2 during the pandemic, this classification proved to be challenging. The uncertainly derived from the lack of reliable and available information on a wide range of essential parameters that affect R0, including, the nature of infection, its duration and severity, transmissibility, infectiousness, the average number of days in which an individual remains infected, population density and health, the average age of the population, the appearance of new variants, and the behavior of the public. In addition, the published Rt and the number of those infected were affected by government measures, the extent of public immunization, the effect of vaccines on the number of new cases and on the number of critically ill patients and the degree of protection against re-infection. Furthermore, the various models examined disease spread on a national level and did not make consider essential variables and unique characteristics to Israel that may influence disease spread, such as its young population, one central entry into the country, emergency preparedness, the population strata, its density, and the number of children per household. Moreover, due to the great complexity of the spread characteristics of the virus, it was not possible to construct a model that included all known variables and the researchers were satisfied with relatively simple models that included using R0 numbers from other countries or calculating R0 numbers according to the number of confirmed infections in Israel with age adjustments. All of these variables created a complex reality that challenged the various models (6). In practice methods that previously helped in predicting disease spread have been found to be less accurate, (7, 8) which has led, among other things, to constant updating of models and methods (9, 10). Over time, each modeling team chose to analyze different data. Although they could better predict the wave trend (increase/decrease), wide gaps remained between the data presented by each model and the actual intensity of morbidity, the number of critically ill patients and mortality. Models that provided accurate predictions for one wave, were very inaccurate in the next wave.

### Public communication

The public's behavior also affected the spread of disease: during the first wave there was great uncertainty together with conflicting reports from sources abroad about a very high R0 number, which contributed to very high public response to the restrictions imposed by the government. As time passed and the public understood that the pandemic may last for a long time, compliance with governmental restrictions decreased, which may have resulted in certain gaps between the models and actual data. For example, while the models predicted a steep rise in morbidity, no such increase was recorded in real life. These gaps may have led to further public non-compliance with governmental restrictions because the predictions did not come true.

The differences between the models' predictions and real-world data shows that policy-making cannot stop the spread of disease altogether, however, it has a limited ability to slow it down, with the intention of trying to flatten its growth curve as much as possible and to avoid very high morbidity in a very short time (and thus to avoid insufficiency of the health system), sometimes even at the cost of dispersing disease spread over a longer period of time.

It is also important to consider the issue of disseminating the models to the public. The extensive media coverage of the COVID-19 pandemic included, among other things, the daily publication of morbidity data alongside various assessments, forecasts and models. Often, this information was partial or was provided without framing the information within the correct context, so that the public only received the "bottom line” (R0 number scenario or prediction of morbidity) without the different parameters that make up the model, the sensitivity tests and parameters that affect the model's accuracy in predicting morbidity, or the difference in the meaning of the scenario (advantages/disadvantages) and its predictions. Due to the recognized importance of models as a tool to help dealing with the pandemic, and the understanding that in the reality of a new disease much more is unknown, it would have been more appropriate to mediate the information in a way that reflected its limitations and shortcomings and to show professional modesty.

Although transparency is a fundamental value in health systems, the damage to public trust that results from publishing information without appropriate mediation and context framing outweighs its benefit. Such damage to trust can lead to decreased public response to professional guidance and to the ability of the government to deal with the spread of disease. In practice, the public's exposure to significant gaps among the scenarios predicted by the models and actual morbidity, increased the public's distrust in decision-makers. Hence, it is very important to mediate the information and the various models to the public in an orderly and reliable manner, while presenting the models correctly and accurately as a tool for decision-making.

### Advice/policy implications

Upon the emergence of a new disease, there is an inherent difficulty in relying on mathematical models as a reliable tool for predicting and formulating a strategy for dealing with its spread. Such uncertainty and a multiplicity of assumptions that are not based on reliable information may lead to significant gaps among the various models, and between the models and real-world data. Data researchers who have agreed to contribute their time and experience toward presenting ways to deal with the COVID-19 pandemic are a welcome phenomenon that should be encouraged and preserved. At the same time, decision-makers must integrate the information presented to them in order to advance knowledge-based decision-making processes on the one hand, but they must also recognize the structural weaknesses of mathematical models when faced with uncertainty. The decision-making process and health policy design should regard models as auxiliary tools and consider their limitations and weaknesses, while remembering that preventive measures, public behavior, seasonality and additional factors influence the models' predictions. Behavioral elements such as public compliance, avoiding crowding, participating in indoor activities, etc. has a large impact on the different models accuracy. Thus, real world data on these parameters can be integrated into the models in order to enhance their precision.

It is essential to correctly mediate the reference scenarios to decision makers and the public, while providing the appropriate context of the mathematical models together with their advantages and disadvantages. In view of the findings of this study, we suggest creating an elaborate mechanism that will serve as a tool for decision-makers. This mechanism should comprise two separate but complementary components: (a) a prediction range derived from combined key models; (b) an independent and separate prediction for each of the models while preserving their different methodologies. Furthermore, we recommend developing a mechanism that would provide modelers access to institutional data in a structured and orderly manner, in addition to the information collected by them independently. This may help to improve and refine the various mathematical models.

## Limitations

First, the current study presents data from different models that were used by policymakers. These models were influenced by several parameters such as policy recommendations, preventative measures, and public awareness, social distancing, etc. this may lead to a potential bias in providing interpretations for the results. However, policy makers used the same models, with the same potential biases, thus we believe the analysis suggested is appropriate. Second, the models were published only in secondary publications and were not peer-reviewed. Nevertheless, these models were presented to the government and health authorities and they served as the basis for decision making.

## Data availability statement

Publicly available datasets were analyzed in this study. This data can be found at: https://datadashboard.health.gov.il/COVID-19/general.

## Author contributions

All authors listed have made a substantial, direct, and intellectual contribution to the work and approved it for publication.

## Conflict of interest

The authors declare that the research was conducted in the absence of any commercial or financial relationships that could be construed as a potential conflict of interest.

## Publisher's note

All claims expressed in this article are solely those of the authors and do not necessarily represent those of their affiliated organizations, or those of the publisher, the editors and the reviewers. Any product that may be evaluated in this article, or claim that may be made by its manufacturer, is not guaranteed or endorsed by the publisher.
